# An Analytical Mobile App for Shared Decision Making About Prenatal Screening: Protocol for a Mixed Methods Study

**DOI:** 10.2196/13321

**Published:** 2019-10-08

**Authors:** Samira Abbasgholizadeh Rahimi, Patrick M Archambault, Vardit Ravitsky, Marie-Eve Lemoine, Sylvie Langlois, Jean-Claude Forest, Anik M C Giguère, François Rousseau, James G Dolan, France Légaré

**Affiliations:** 1 Department of Family Medicine McGill University Montreal, QC Canada; 2 Lady Davis Institute for Medical Research Jewish General Hospital Montreal, QC Canada; 3 Department of Family Medicine and Emergency Medicine, Faculty of Medicine, Université Laval Québec, QC Canada; 4 Centre de recherche Centre intégré en santé et services sociaux de Chaudière-Appalaches Lévis, QC Canada; 5 Centre de recherche sur les soins et les services de première ligne de l’Université Laval Université Laval Québec, QC Canada; 6 Programmes de bioéthique, Département de médecine sociale et préventive, École de santé publique de l'Université de Montréal, Université de Montréal Montréal, QC Canada; 7 Department of Medical Genetics, University of British Columbia Vancouver, BC Canada; 8 Centre de recherche, Centre hospitalier universitaire de Québec Québec, QC Canada; 9 Department of Molecular Biology, Medical Biochemistry and Pathology, Faculty of Medicine, Université Laval Québec, QC Canada; 10 Canadian Research Chair in Shared Decision Making and Knowledge Translation Québec, QC Canada; 11 Department of Public Health Sciences, University of Rochester Medical Center Rochester, NY United States

**Keywords:** shared decision making, multiple criteria decision analysis, analytic hierarchy process, decision aid, prenatal screening, mobile app

## Abstract

**Background:**

Decisions about prenatal screening to assess the risk of genetic conditions such as Down syndrome are complex and should be well informed. Moreover, the number of available tests is increasing. Shared decision making (SDM) about testing could be facilitated by decision aids powered by mobile technology.

**Objective:**

In this mixed methods study, we aim to (1) assess women’s needs and preferences regarding using an app for considering prenatal screening, (2) develop a decision model using the analytical hierarchy process, and (3) develop an analytical app and assess its usability and usefulness.

**Methods:**

In phase 1, we will assess the needs of 90 pregnant women and their partners (if available). We will identify eligible participants in 3 clinical sites (a midwife-led birthing center, a family practice clinic, and an obstetrician-led hospital-based clinic) in Quebec City and Montreal, Canada. Using semistructured interviews, we will assess participants’ attitudes toward mobile apps for decision making about health, their current use of apps for health purposes, and their expectations of an app for prenatal testing decisions. Self-administered questionnaires will collect sociodemographic information, intentions to use an app for prenatal testing, and perceived importance of decision criteria. Qualitative data will be transcribed verbatim and analyzed thematically. Quantitative data will be analyzed using descriptive statistics and the analytic hierarchy process (AHP) method. In phase 2, we will develop a decision model using the AHP whereby users can assign relative importance to criteria when deciding between options. We will validate the model with potential users and a multidisciplinary team of patients, family physicians, primary care researchers, decision sciences experts, engineers, and experts in SDM, genetics, and bioethics. In phase 3, we will develop a prototype of the app using the results of the first 2 phases, pilot test its usefulness and usability among a sample of 15 pregnant women and their partners (if available), and improve it through 3 iterations. Data will be collected with a self-administered questionnaire. Results will be analyzed using descriptive statistics.

**Results:**

Recruitment for phase 1 will begin in 2019. We expect results to be available in 2021.

**Conclusions:**

This study will result in a validated analytical app that will provide pregnant women and their partners with up-to-date information about prenatal screening options and their risks and benefits. It will help them clarify their values and enable them to weigh the options to make informed choices consistent with their preferences and values before meeting face-to-face with their health care professional. The app will be easy to update with the latest information and will provide women with a user-friendly experience using their smartphones or tablets. This study and the resulting app will contribute to high-quality SDM between pregnant women and their health care team.

**International Registered Report Identifier (IRRID):**

DERR1-10.2196/13321

## Introduction

### Prenatal Testing

Prenatal screening for trisomies 21 (Down syndrome), 18 (Edwards syndrome), and 13 (Patau syndrome) and for open neural tube defects has been offered to expecting women and couples for 3 decades. This type of screening differs from most disease screening programs in that it is not promoted as a public health means to reduce the incidence of the detected conditions. It is rather construed as a means to promote the reproductive autonomy of pregnant women and their partners, aiming to provide them with information that may be relevant to their reproductive decision making. Such decisions would be based on their knowledge about the fetus as well as their values and preferences [1]. Hence, prenatal screening is not meant to be a tool for promoting pregnancy termination for affected fetuses. Many pregnant women and couples choose to terminate pregnancies when the fetus is diagnosed with a trisomy [2], and some advocacy groups express concerns about screening contributing to stigmatization of disabled individuals [3]. Prenatal screening tests indicate a probability of the fetus having a given condition, but a diagnostic test is needed to confirm the result. Diagnostic tests such as amniocentesis and chorionic villi sampling do provide definitive results but entail a small risk of miscarriage. Thus, prenatal screening may reduce the number of invasive tests and their associated pregnancy losses. Testing also enables couples to prepare for the birth of a child with special needs, who is likely to benefit from specialized pregnancy follow-up and specialized care at birth and beyond [4].

However, the decision to undergo the initial probability screening leads to furthermore challenging decisions. Positive screening results lead to a further decision about the more invasive diagnostic testing. If this diagnostic result is positive, women face an even more difficult decision: whether to terminate the pregnancy or not. Some people prefer not to engage in this decision-making process in the first place. Reasons for declining screening include a preference for a less medicalized way of experiencing the pregnancy; an ideological, political, or religious opposition to screening; accepting the risks of having a child with the conditions tested for; preferring not to know in advance; and practical constraints [5-7]. Therefore, the option of choosing to decline screening must be available. Pregnant women and their partners must be adequately informed about the risks (or potential future risks) and benefits of screening tests and understand that such tests are optional and that they will be fully supported regardless of the path they choose [8].

### Shared Decision Making and Multiple Criteria Decision Analysis

Many women and their partners are unaware of the implications of embarking on the path of prenatal testing [9]. The complexity of the testing decisions requires that the offer of testing be accompanied by (1) neutral, balanced, and comprehensive information on testing options and the conditions that are being detected, and (2) a space for making these very personal decisions in a way that is free of undue influence [9-11]. This reflects the fact that informed choice and free choice are basic components of an autonomous decision.

Women and their partners can be accompanied in this way through shared decision making (SDM), a process by which patients and clinicians collaborate to make decisions based on accurate information and on what matters most to them [12]. Decision aids (DAs) are SDM tools that can take various forms (such as a brochure, a booklet, an app, or a Web page). They provide information and solicit patients’ values and preferences [13]. A Cochrane systematic review showed that DAs increase participants’ knowledge, the accuracy of their risk perceptions, and the match between their values and the option chosen. In addition, it showed that DAs decrease decisional conﬂict (or discomfort) relating to feeling uninformed, decrease indecision about personal values, and decrease the proportion of people who are passive in the decision-making process [13]. Therefore, SDM and DAs work well with the reproductive autonomy rationale underlying prenatal screening, and SDM has been identified by patients as a preferred approach to guide decision making.

Once women and their partners have made a decision to undergo prenatal screening, they must choose from an increasing number of screening options and consider multiple and often conflicting decision criteria [14,15]. For example, the risk of false positives (false alarm) or false negatives (false reassurance) for each test, the longer or shorter wait times for the results and the variable costs and coverage schemes. In addition to best evidence about risks and benefits, decisions need to be informed by what couples consider most important, that is, their preferences and values. For instance, a test involving new technology may be costly but may give the results in less time and with higher accuracy than one covered by insurance. Thus, couples need to decide whether finding out more accurate results and sooner is more important to them than the cost of the test, and if so, how much more important.

People usually have difficulty in making decisions when they face complex problems with multiple options involving value-based trade-offs between the advantages and disadvantageous of each option [16]. Multiple criteria decision analysis (MCDA) is an umbrella term for a number of methods for evaluating multiple conflicting criteria in decision making (both quantitative and qualitative criteria). These methods could help women decide among the multiple options for prenatal screening based on their values [17]; however, to the best of our knowledge they never been used in this context.

The analytic hierarchy process (AHP) is one MCDA method that has been used successfully (alone or integrated with other methods) in numerous medical decision-making contexts and in complex circumstances such as medication decision making in type 2 diabetes [18], priorities regarding colorectal cancer screening [19], prioritizing orthopedic patients for elective surgery (integrated with other methods) [20], and for prioritization of organ transplant patients [21]. The steps of the AHP match well with the essential elements of SDM. The SDM steps are as follows: define the problem and options available, review pros and cons of options, elicit patient values and preferences, clinician recommendations, review patient’s ability to implement plan, check understanding, and make or defer the decision [22]. For example, for reviewing the pros and cons of the various tests, the corresponding AHP step is to make pairwise comparisons about how well the options satisfy the decision criteria; and for the SDM step of eliciting patient values and preferences, the AHP step is to make pairwise comparisons to prioritize value-based criteria affecting the decision [17]. The AHP is a quantitative technique but it can consider both quantifiable and nonquantifiable criteria. It is methodologically sound, systematic, and user friendly. It frames a decision as a hierarchy, which makes it easy to explain, and all inputs consist of consecutive comparisons of pairs of decision elements (eg, decision criteria and options). These *pairwise comparisons* are considered to be one of the best ways to elicit judgments from people [17] and seem well suited to app-based DAs.

Recent studies indicate an increase in the use of mobile phones and other wireless technology for health care purposes (mobile health [mHealth]) [23]. More than 85% of clinicians are now owners of smartphones and approximately 50% of them use smartphone apps in their clinical practice [24]. In the United States, a nationwide survey in 2012 reported that 33% of cellphone owners used mobile phones for health information, whereas 2 years earlier this was only 17% [25].

Even though mHealth tools cannot replace face-to-face communication with health care professionals during a consultation, as a form of DA they can be an important complementary and support tool. A well-designed app for health care purposes is accessible, has easy-to-follow procedures, and can automatically integrate the latest medical evidence. This could support SDM, improve clinical outcomes, and result in positive lifestyle changes [26,27].

### Context

In the province of Quebec, Canada, pregnant women are followed up in hospital obstetrics departments, birthing centers, and family practice groups and are routinely offered 2 Down syndrome prenatal screening tests: (1) the serum integrated prenatal screening (SIPS), involving 2 blood tests (at 10-13 weeks, then at 14-16 weeks); (2) integrated prenatal screening (IPS), involving the same 2 tests, plus a nuchal translucency test based on a fetal ultrasound (11-14 weeks). Noninvasive prenatal testing (NIPT), or cell-free fetal DNA screening, which involves 1 blood test at 9 weeks and offers a higher level of reliability, is not yet covered by provincial health insurance in Quebec but can be purchased privately. For women whose SIPS or IPS results show a high risk of bearing a child with Down syndrome, public insurance will cover the NIPT test as of 2019. If these test results are positive, patients are offered amniocentesis.

Earlier, we developed a paper-based DA following a rigorous procedure [28] and subsequently updated it with the latest tests and an innovative decision model [29]. For the update, first we looked for recent evidence on prenatal screening and considered other prenatal screening DAs that included the new NIPT test. The development team reached consensus on which of the latest evidence should be considered. Data were updated, added, or removed from the DA in consequence, and a new selection hierarchy was incorporated to facilitate patients’ selection of screening tests. The updated DA obtained a score of 16 out of 16 on the International Patient Decision Aids Standards checklist (Multimedia Appendix 1). Finally, the development team reached consensus on the final updated version of the DA. Usability, usefulness, and acceptability of this DA have been evaluated in another project with 45 couples in Quebec City, Canada.

The overarching aim of this study is to empower pregnant women and their partners with mobile technology so they can make informed decisions about prenatal screening with the support of their health care team. Thus, our specific objectives are to (1) assess the needs and preferences of pregnant women regarding the use of an app for deciding about prenatal screening, (2) develop a decision model using the AHP, and (3) develop an analytical app and assess its usability and usefulness.

## Methods

### Study Design

**Figure 1:**
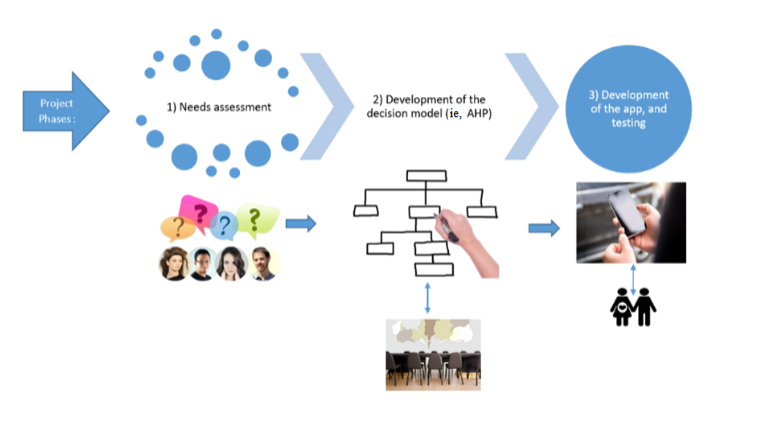
Road map of the project. AHP: analytic hierarchy process.

We propose a multipronged mixed methods study design in 3 phases: (1) needs assessment, (2) decision model development, and (3) analytical app development and pilot testing for usefulness and usability (Figure 1). As health care in Canada, including prenatal screening programs, is delivered under provincial and territorial rather than federal health insurance plans, we will focus on the province of Quebec. Quebec is a largely French-speaking province in Eastern Canada with over 8 million inhabitants. We will conduct our study in Quebec City and Montreal.

This project will be guided by a multidisciplinary steering committee of experts in SDM (FL, SAR, AG, and PA), family medicine and primary care (FL), prenatal care and genomics (FR, JCF, SL, and VR), engineering and technology (SAR), bioethics (VR), knowledge translation (FL, AG, PA, and SAR), and decision sciences and MCDA methods (SAR and JD). Patient representatives (pregnant women or women who have experienced pregnancies and their partners) will be involved in each phase to validate the decision model, determine the content of the app, and design it. The study has been approved by the ethics committee of the Population Health and Primary Care Research Division of the Centre intégré universitaire de santé et de services sociaux de la Capitale nationale (#2019-1534).

### Phase 1: Needs Assessment

#### Participants

Using purposeful sampling, we will recruit a sample of 15 potential end users of the app (women and their partners if available), in each of the 3 clinical sites (midwife-led birthing centers, family practice clinics, and obstetrician-led hospital-based clinics) in Quebec City and Montreal for a total of 90 participants. An experienced research assistant and a trainee or 2 research assistants will meet pregnant women in the waiting rooms at each of these sites before their prenatal care appointment and invite those who are eligible to participate in the study. We expect to achieve theoretical saturation [30]. To be eligible to participate in both phases 1 and 3 of our study, pregnant women must (1) be at least 18 years old, (2) be more than 20 weeks pregnant or have given birth during the previous year (so as not to interfere with a decision about a current pregnancy), (3) have a low-risk pregnancy (ie, no complications such as hypertension or diabetes), (4) not be expected to give birth close to the data collection dates, (5) be able to speak and write either French or English, and (6) be able to give informed consent. Pregnant women’s partners (if available and interested in participating in the study) will be asked to give informed consent. No eligibility criteria will be applied to the partners. If just 1 member of the couple but not both give informed consent, only the partner who gave consent will be interviewed. Pregnant women who are single are also eligible.

We chose these sites because all prenatal follow-up in the province of Quebec takes place in these 3 types of site. In addition, each attracts somewhat different clienteles, and we wanted to ensure that we include the needs and perspectives of as broad a spectrum of socioeconomic, ethnic, and linguistic communities (eg, immigrants and Anglophones) as possible in our study. We will recruit a minimum of 9 out of 45 participants from diverse ethnic and linguistic communities in Montreal and a minimum of 2 out of 45 in Quebec City to reflect the respective proportions of these population in the 2 cities [31].

#### Data Collection

Informed by recommendations about how to evaluate eHealth tools [32], and a study on needs assessments for mobile technology [33], we developed an interview guide to conduct semistructured interviews with couples or single participants to assess (1) their attitudes toward mHealth apps in general and for prenatal screening decisions in particular; (2) their current use (if any) of apps for the purpose of information seeking about health and decision making; (3) their opinions, suggestions, and preferences regarding an app for decision making about prenatal screening such as the one we are proposing.

Two trained research assistants or a research assistant and a trainee with expertise in health care research will conduct the interviews. No previous relationship will exist with participants other than a call or email contact to set a date for the interview.

According to participants’ preferences, interviews will be conducted in a research center, in one of the recruitment sites, or in any other place convenient for them. Interviews will be conducted in French or English. Interviews are expected to last about 60 to 90 min. Written informed consent will be obtained at the beginning of the interview.

**Figure 2:**
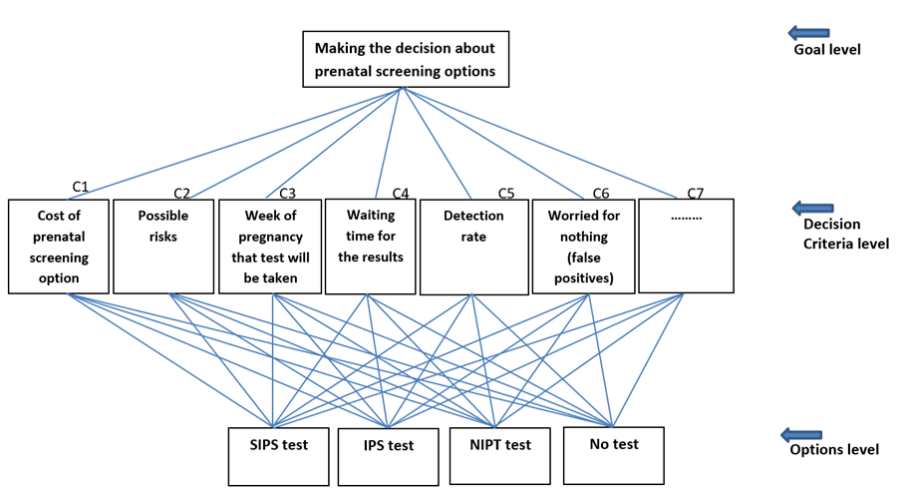
Preliminary decision model. IPS: integrated prenatal screening, NIPT: noninvasive prenatal testing, SIPS: serum integrated prenatal screening.

At the end of the interview, each participant or couple will be invited to fill out a self-administered questionnaire, validated by experts in the field, to (1) evaluate their intention to use an app for decision making about prenatal screening and (2) answer a series of questions (based on the AHP method) comparing the relative importance of various predefined decision criteria (see Figure 2) [29]. They will also complete a sociodemographic questionnaire.

#### Data Analysis

Interviews will be audio-recorded and transcribed verbatim. We will perform a thematic analysis while taking into account emerging themes. Two authors will separately code the transcripts using the NVivo qualitative data analysis software (QSR International Pty Ltd. Version 12, 2018). They will cross-check their codes and categorize the themes. They will then produce a report on the most relevant categories and an overview of the most important preferences, for example, features of the app that participants would like to see in a prototype (for phase 3).

Quantitative data will be analyzed using descriptive statistics. The AHP will be used to analyze the relative importance of decision criteria. We will adapt the CPD-Reaction (Continuing Professional Development Reaction) questionnaire, developed by our team to assess behavioral intention, to evaluate the intention of participants to use an app to decide about prenatal screening [34,35]. We will summarize sociodemographic characteristics and variables and compute scores from the adapted CPD-Reaction questionnaire; descriptive statistics such as mean, standard deviation, and median will be calculated for continuous data and frequencies for categorical data. The intention scores will be computed using the mean of the 2 intention items of the adapted CPD-Reaction. The reliability of the intention construct will be confirmed on these 2 items by Cronbach alpha testing with a level of statistical significance of less than .05. We will also perform bivariate analyses and multivariate analyses to assess participants’ intentions to use an app for decision making. All variables will be entered in the multivariate model using the backward elimination procedure to obtain an adjusted model with better goodness of fit.

The example of questions related to the AHP are provided in Multimedia Appendix 2. The responses to these questions will be exported to a Microsoft Excel spreadsheet and will be analyzed using the AHP method explained below.

### Phase 2: Development of the Decision Model Using the Analytic Hierarchy Process Method

In this phase, we will develop a decision model using the AHP method, based on the results of phase 1 and the content of our validated paper-based DA for prenatal screening [29] (Multimedia Appendix 1). The decision model will be guided by the routine prenatal screening procedure in the province of Quebec, Canada [36,37]. The 6 AHP steps suggested by Dolan et al for performing the AHP method to promote SDM [17] and Saaty’s AHP [38] will be followed during the development of the decision model and its implementation in our app prototype. The 6 steps are described in the following sections.

#### Step 1: Define the Decision Elements and the Working Knowledge Base

This step consists of the following:

1. Defining the elements of the decision. The elements are the goal and decision, the options to be considered, and the criteria that will be used to determine how well the options meet the goal.
2. Examining the working knowledge base to see if there is a dominant option that is clearly better than the others across all of the decision criteria.

This first step was already accomplished during the development of the paper-based DA [29] (Multimedia Appendix 1).

#### Step 2: Construct the Decision Model

The second step is to arrange the decision elements into a hierarchy. The prototype of the model for prenatal screening is shown in Figure 2. The goal is at the top, making a decision about prenatal screening; the options are at the bottom (SIPS, IPS, NIPT, and no test); and the decision criteria are in between.

#### Step 3: Divide the Decision into Smaller Parts and Make Pairwise Comparisons to Determine Local Priorities

In the AHP approach, the decision is analyzed by dividing it into smaller parts and creating comparison pairs between decision elements on lower levels of the hierarchy relative to each element on the next higher level [16]. In other words, decision options are compared relative to each criterion. Every possible pair will be compared. Comparisons can be verbal, numerical, or graphic. In this study, we will use the numerical comparison format, Saaty’s 9-point scale [38]. Users will first be asked if the 2 elements being compared are equally important or preferable relative to the referent element on the next higher level of the hierarchy or if one is more important or preferable than the other. If they are equal, no further input is needed. If one is more important or preferable, the decision maker indicates how much more important or preferable on a scale of 1 to 9. For example, they will indicate if cost is more important to them than time or vice versa. After all the comparisons are completed, a comparison matrix will be created and then, using matrix algebra procedures, ratio scales will be created. These scales indicate the relative priorities of the criteria in meeting the goal and the priorities of each option considering each criterion [16,38].

#### Step 4: Synthesis

In this step, all of the scales created in step 3 will be combined to determine how well the options (ie, SIPS, IPS, NIPT, and no test) will meet the goal: an informed decision about prenatal screening that reflects patients’ preferences. This can be done through either distributive synthesis, which ranks the options in order of preference, or ideal synthesis, which identifies a single best option. We will choose the most appropriate synthesis based on users’ needs.

#### Step 5: Sensitivity Analysis

This is an optional but useful step. Sensitivity analyses will help determine how sensitive the selected option will be by changing one input while keeping the other inputs constant. It could help determine how the different judgments of the patients affect the analysis. The possibility of adding this step will be evaluated with expert team members and patient partners.

#### 

The final step is to either make the prenatal screening decision or go back and refine the analysis until a decision can be made. We will integrate the 4-item SURE (Sure of myself; Understand information; Risk-benefit ratio; Encouragement) screening test designed to screen for clinically significant decisional conflict [39] into our analytical app. If the pregnant women and their partners are not sure of their decision, they can go back and reconsider their options.

The steps of the model will be added to the app. Pregnant women and their partners will use this app to weight decision options based on their preferences and on what is most important to them, compare the available prenatal screening options in the light of these preferences, and finally select one. The decision model will be validated, after development, with a team of experts and patient partners in a meeting.

### Phase 3: Development of the Analytical App and Pilot Testing

#### Development of the App

In this phase, we will employ a user-centered iterative approach to developing the mobile app. The app will be for mobile devices or tablets using leading operating systems such as Android and iOS. It will be written in the official development language of these operating systems. Users will not need access to the internet after downloading the app, except when updates become available. The app prototype will be developed in cooperation with a partner commercial company and in regular consultation with the patient partners.

The app will be divided into 4 main parts: (1) the information needed to make the decision, including the information on trisomy 21 (T21), trisomy 18 (T18), and trisomy 13 (T13), and the 4 different prenatal screening options (ie, SIPS, IPS, NIPT, and no screening), (2) the information on advantages and disadvantages of *doing the test* and *not doing the test*, (3) the AHP pairwise comparisons to weigh the criteria leading to a decision (advantages and disadvantages of each test and preferences), and (4) the SURE screening test to make sure the user is sure about their decision [39]. We will ensure the features of the app respond to the needs expressed in phase 1. This will be accomplished at meetings attended by the multidisciplinary expert team and the technology company.

#### Pilot Testing

After development of the first prototype, we will conduct pilot testing to evaluate the perceived usability and usefulness of the app and improve it iteratively. We will recruit a sample of 15 pregnant women and their partners (if available) consulting for prenatal care in the same 6 sites as in phase 1. Participants will be couples or individuals who participated in phase 1 and new participants will be recruited if needed to reach the targeted sample size. Participants will be invited by personal email or phone or in person at the clinical sites. The same eligibility criteria as in phase 1 will apply.

Participants will be invited to use the app and give feedback. As this phase will be iterative, we will start by recruiting 5 couples. During semistructured interviews, lasting 60 to 90 min, we will ask them to assess the usability and usefulness of the prototype app based on a self-administered questionnaire, and then we will ask them for their suggestions for its improvement. Usability will be assessed using the system usability scale (SUS) [40]. SUS is frequently used to measure user experience and utility of information systems, including the efficacy and satisfaction with which users accomplish specific tasks. This scale comprises 10 statements that assess participants’ immediate reaction to the use of a technology before any discussion with the researcher. The scale is also used to explore users’ needs regarding a prototype or to evaluate the usability of an existing technology. Users will assess usefulness on a scale of 1 to 5. In addition, we will use the 10 items of the Preparation for Decision Making scale [41] to assess how useful the app is for preparing a respondent to communicate with their health professional at a consultation focused on making a prenatal testing decision. Participants’ feedback in this first wave will be used to improve the app prototype, and then we will move into recruiting the next waves of 5 couples or individuals, repeating the same data collection process. In keeping with the literature and our previous work [42], we expect to reach saturation with a maximum of 3 waves of feedback [30].

## Results

For phase 1 of this project (needs assessment), recruitment will begin in 2019. Recruitment and data collection will continue until 90 participants (45 in Quebec City and 45 in Montreal) have been interviewed. The results of phase 2 will lead to the development of the analytical decision model. The results of phases 1 and 2 will be integrated into phase 3 to develop the app. Following this step, the app will be pilot tested. All 3 phases of the study are expected to be completed in 2021. The reporting of results will follow the mHealth evidence reporting and assessment checklist [43], and the reporting of qualitative results will also follow the COnsolidated criteria for REporting Qualitative research guideline [44].

## Discussion

### Overview

By the end of the study, we will have developed and validated an analytical app that will provide pregnant women and their partners with the most up-to-date information about the various prenatal screening options and their risks and benefits, including the option of no testing. We expect this app to inform pregnant women and their partners and help them to consider various decision criteria in the light of the most recent information and their values to make a decision that they are comfortable with and hence provide truly informed consent to screening. It will enable them to rate the importance of criteria to be considered and finally share a decision consistent with their preferences and values with their health care professional. As with other forms of DA, health care providers can either accompany women and their partners through the process of using the mobile app or they can discuss the decision afterwards when the decision process is still fresh in the minds of their patients. The app will be user-friendly and easy to update with the latest information.

Finally, the proposed AHP method has a solid methodological base and is easy to use and integrate into SDM tools such as DAs to contribute to SDM. AHP can facilitate eliciting patient values and preferences, and we believe it is ideally suited to help pregnant women and their partners with various levels of health literacy to process this complex decision.

### Strengths and Limitations

Our team has 15 years of experience in the development of shared decision-making tools and has been working on tools specifically for prenatal testing decisions for more than 5 years. To the best of our knowledge, the AHP approach has never been used in this context. Developing the AHP model will provide a systematic approach for helping pregnant women and their partners choose whether to undergo prenatal testing or not, and if so, what options match their preferences and values. Using pairwise comparisons, the AHP enables patients to consider both quantifiable (objective considerations such as cost and time) and nonquantifiable criteria (subjective considerations such as women’s feelings and values) in the analysis. This method could be applied to other complex decisions.

In terms of limitations, recruiting 90 pregnant women with or without their partners in 3 different sites in 2 cities (Quebec City and Montreal) may be challenging. Second, the study targets couples in the province of Quebec, that is, in a single health care system, so we cannot infer that our results are applicable to other populations. Finally, enabling women and their partners to fully share their decision-making process with their health care provider after using the app may require further study.

## Appendix

Multimedia Appendix 1 Decision Aid.

Multimedia Appendix 2 Questionnaire.

## Abbreviations

AHP: analytic hierarchy process
CPD: continuing professional development
DA: decision aid
IPS: integrated prenatal screening
MCDA: multiple criteria decision analysis
mHealth: mobile health
NIPT: noninvasive prenatal testing
SDM: shared decision making
SIPS: serum integrated prenatal screening
SURE: Sure of myself; Understand information; Risk-benefit ratio; Encouragement
SUS: system usability scale
