# Supplementary material for: An Analytical Mobile App for Shared Decision Making About Prenatal Screening: Protocol for a Mixed Methods Study
Source: JMIR Res Protoc. 2019 Oct 8;8(10):e13321. doi: 10.2196/13321 (PMC6913686; doi:10.2196/13321)
Supplement: Multimedia Appendix 1 [file resprot_v8i10e13321_app1.pdf]

Appendix 1

Decision Box
PATIENT
...to help you make a decision

What are my options regarding prenatal screening tests?

► *Who might consider being screened?*

All pregnant women can choose to have screening. The risk of Trisomy 21 (T21), Trisomy 18 (T18) and Trisomy 13 (T13) increases with the woman's age.

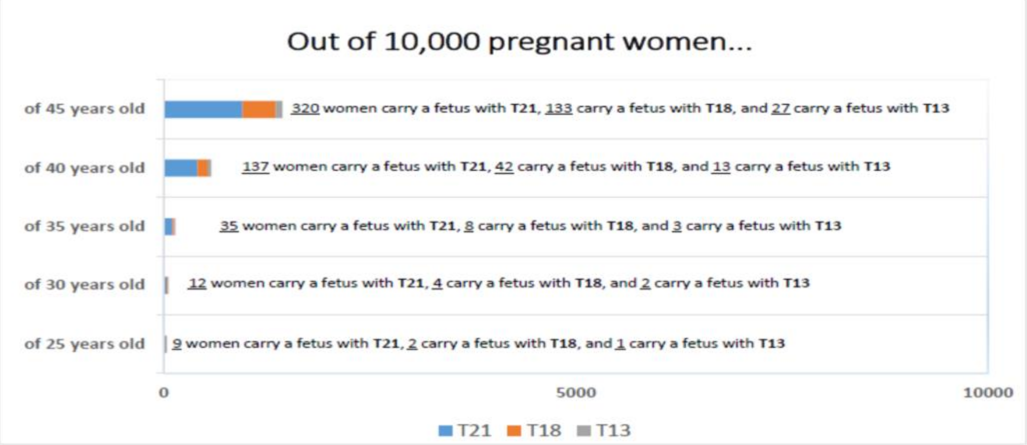

What is Trisomy 21 (or Down syndrome)?

- It is caused by having an extra copy of chromosome 21.
- People with T21 have intellectual disabilities that vary from slight to moderate, but are sometimes serious, with poor muscle tone, very supple joints, greater risk of vision, hearing defects, cardiac and/or gastro-intestinal defects.
- 60% of children with T21 require specialized home care.
- Some adults with T21 have jobs and are almost completely independent.
- People with T21 can develop meaningful emotional relationships and fulfilling lives for themselves, their families and friends.

What are Trisomy 18 and Trisomy 13?

- T18 and T13 are caused by having an extra copy of chromosome 18 or 13.
- Many pregnancies with T18 and T13 will be miscarried or result in the fetus dying in utero.
- Babies born with T18 and T13 rarely live more than a few months because of serious heart and brain defects, and poor growth before and after birth.

A decision to make:

➢ *Doing and not doing the test are both acceptable choices. We suggest that you:*
  - Base your decision on the best scientific evidence and on your values and preferences.
  - Share the decision with your health professional, and partner.

© Université Laval, 2017. All rights reserved

➢ *What are my options for prenatal screening tests?*

My options:

Serum Integrated Prenatal Screening (SIPS) test

- Two maternal blood tests. First blood test between 10 - 13 weeks and second blood test between 14 - 16 weeks.
- Waiting time for results is 10 days after second blood test.
- Detection rate is 85 per 100 cases of T21 (85%) amongst women who get a result.
- The cost is covered by RAMQ in the province of Quebec.

Integrated Prenatal Screen (IPS) test

- Same two maternal blood tests as SIPS, and an ultrasound of the back of the fetus neck (NT scan) between 11-14 weeks.
- Waiting time for results is 10 days after second blood test.
- Detection rate is 90 per 100 cases of T21 (90%) amongst women who get a result.
- The cost is covered by RAMQ in the province of Quebec.

Non-Invasive Prenatal Test (NIPT)

- One maternal blood test. From 9 weeks of pregnancy.
- Waiting time for results is 10 days after the blood test.
- Detection rate is 99 per 100 cases of T21 (99 %) amongst women who get a result *.
- The cost is up to $800 in private clinics.

No test

- No test.
- No waiting.
- Will not be detected.
- No cost.

For SIPS, IPS and NIPT:

- **If the test says the risk is high,** an amniocentesis will be offered as a diagnostic test.
- ***What is amniocentesis?*** An amniocentesis is a diagnostic test that checks the chromosomes of fetuses considered to be at risk of specific problems such as Down syndrome.
  A small sample of the liquid surrounding the fetus is taken using a needle inserted through the mother's abdomen into her uterus under continues ultrasound guidance.

For No test:

- See benefits and harms of not doing the test on page 4.

*About 5% of women who take the NIPT will not have results on the first try, and will have to redo the test or take a different screening test.

© Université Laval, 2017. All rights reserved

Multimedia Appendix 1

An Analytical Mobile App for Shared Decision Making About Prenatal Screening: Protocol for a Mixed Methods Study

➢ What can happen after the prenatal screening test?

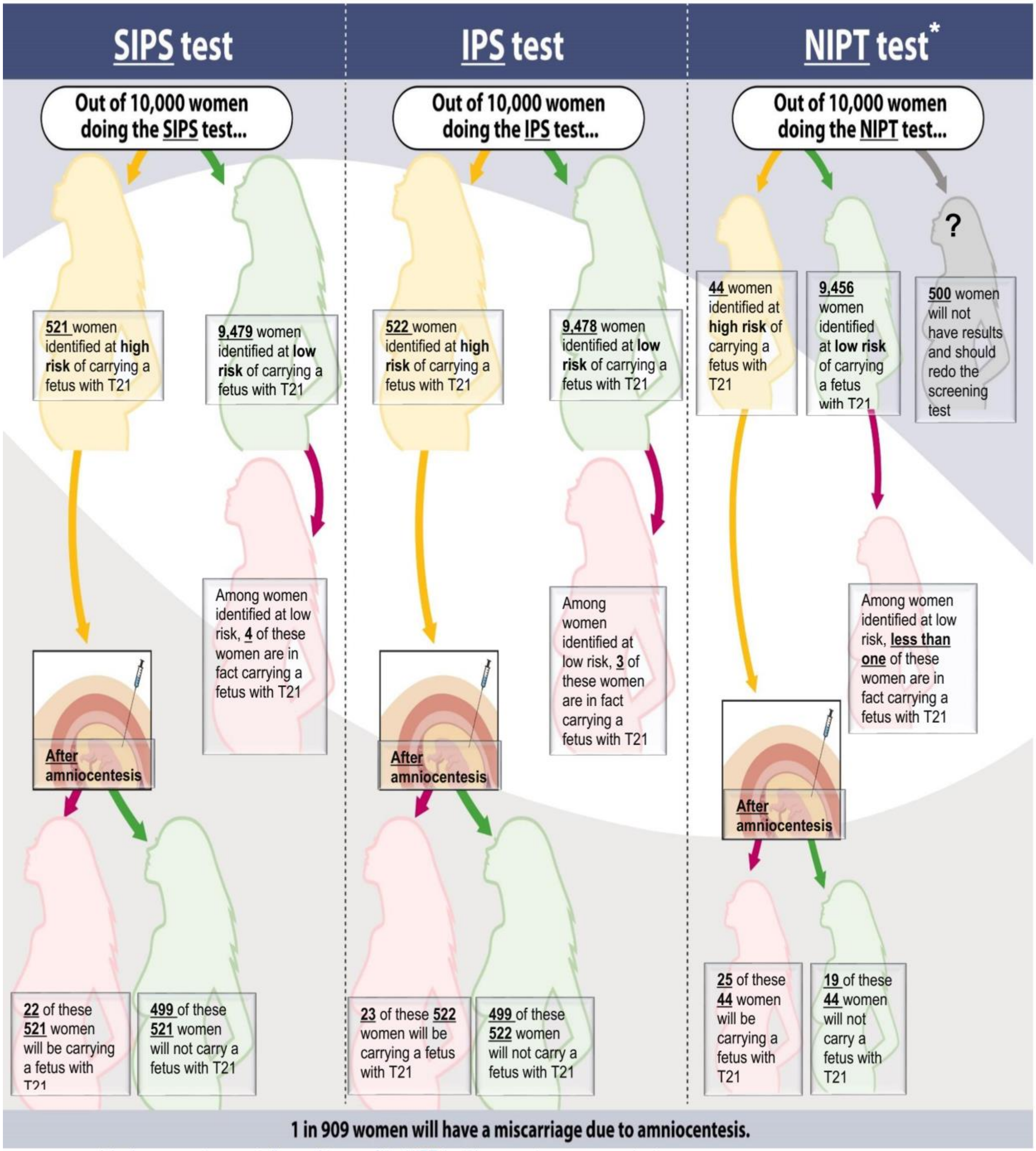

* As there are not many studies on this use of the NIPT test, these numbers are approximate.

© Université Laval, 2017. All rights reserved

➢ *What concerns me about the benefits and harms of doing the test and not doing the test?*

1. Review the possible benefits and harms of each test. Add any other benefits and harms that are important to you.
2. Show how important each benefit and harm is to you by circling the stars (5=very important, 1=not important).

Benefits and harms of doing the prenatal screening test:

Benefits: — How important is it to you?

- **Reassurance** — * * * * *
  For example, out of 10,000 women who take the NIPT test, 9,456 are identified at low risk of carrying a fetus with T21. These women are reassured.
- **Prepare to end the pregnancy or to have a child with T21** — * * * * *
  Among the women who know they are carrying a fetus with T21, those who choose to continue the pregnancy can prepare for a child with T21.
- **Knowing your chances of carrying a fetus with T21** — * * * * *
  For example, out of each 10,000 women screened, 44 are identified by NIPT test as being at high risk of carrying a fetus with T21. If these women have an amniocentesis to verify the results of the screening, 25 would actually be carrying a fetus with T21.
- **Other benefits: ....** — * * * * *

Harms: — How important is it to you?

- **False reassurance** — * * * * *
  For example, 3 of the 9,478 women identified by IPS test as at low risk of carrying a fetus with T21 will actually be carrying a T21 fetus. These women will have been falsely reassured.
- **False alarm** — * * * * *
  For example, out of the 522 women identified by IPS test as being at high risk of carrying a fetus with T21, 499 are not actually carrying a fetus with T21. Many of these women experience anxiety. 1 in 909 will have a miscarriage as a result of the amniocentesis to verify screening test results.
- **Anxiety while waiting for results** — * * * * *
  Based on previous studies, child-related anxiety levels were higher in women who chose to be screened compared to women who declined screening.
- **Other harms: ....** — * * * * *

Benefits and harms of not doing the prenatal screening test:

Benefits: — How important is it to you?

- **Avoid an unnecessary test** — * * * * *
  Out of 10,000 women doing IPS test, 9,478 are not carrying a fetus with T21. By not doing the test, 499 of the 522 pregnant women (identified by IPS test as being at high risk of carrying a fetus with T21) will avoid an unnecessary amniocentesis, and 1 in 909 women will avoid a miscarriage caused by the amniocentesis.
- **Stay true to your personal convictions** — * * * * *
  For some women, not doing the test is in keeping with their personal convictions.
- **Avoid anxiety and avoid difficult decisions such as whether to end the pregnancy** — * * * * *
  Women who do not take the test avoid the anxiety of:
  - waiting for the test results.
  - making a decision about whether to do the amniocentesis if the test shows a high risk of T21.
  - making a decision about ending the pregnancy.
- **Other benefits: ....** — * * * * *

Harms: — How important is it to you?

- **Not knowing your risk of carrying a fetus with T21** — * * * * *
  For example, out of 10,000 women who do not take the IPS screening test, 23 are carrying a fetus with T21.
  These women cannot prepare for living with a child with special needs. They may regret not having done the prenatal screening test.
- **Anxiety about the outcome of the pregnancy** — * * * * *
  Women who don't take the prenatal screening test may be anxious because they don't know if their child will have T21 or not.
- **Other harms: ....** — * * * * *

➢ What is your decision?

Do the test ☐ Not doing the test ☐

If you chose "Do the test" go to the **page 5** and choose the test. If you chose "Not doing the test", go to the **page 6**.

© Université Laval, 2017. All rights reserved

➢ *Which prenatal screening test should I choose?*

The blue boxes on the left show the concerns about the tests that may be important to you. You can add other concerns in the last box depending on your values or opinions.

1) Show how important each concern is to you by circling the stars (5 = very important, 1 = not important).
2) Considering the importance of each of your concerns, select the best test in the righthand column.
3) Choose the test which is best for you.

Different concerns about prenatal screening tests

		1) Importance of this concern	2) Considering this concern, which test(s) do you prefer?
Week of pregnancy that test will be taken	SIPS: 1st blood test in 10 - 13 weeks and 2nd blood test in 14 - 16 weeks of pregnancy.<br>IPS : 1st blood test in 10 - 13 weeks , 2nd blood test in 14 - 16 weeks, and NT scan in 11-14 weeks of pregnancy.<br>NIPT: From 9 weeks of pregnancy.	★★★★★	SIPS ☐<br>IPS ☐<br>NIPT ☐
Waiting time for the results	SIPS: 10 days after second blood test.<br>IPS : 10 days after second blood test.<br>NIPT: 10 days after blood test.	★★★★★	SIPS ☐<br>IPS ☐<br>NIPT ☐
Detection rate of each test	SIPS: 85 per 100 cases of T21 (85%) among women who do the test.<br>IPS: 90 per 100 cases of T21 (90%) among women who do the test.<br>NIPT: 99 per 100 cases of T21 (99%) among women who get results.	★★★★★	SIPS ☐<br>IPS ☐<br>NIPT ☐
Worried for nothing (risk of a false positives)	SIPS: About 500 per 10,000 pregnancies.<br>IPS: About 500 per 10,000 pregnancies.<br>NIPT: About 19 per 10,000 pregnancies.	★★★★★	SIPS ☐<br>IPS ☐<br>NIPT ☐
Cost of each test	SIPS: Covered by RAMQ in province of Quebec.<br>IPS: Covered by RAMQ in province of Quebec.<br>NIPT: Up to $800 in private clinics.	★★★★★	SIPS ☐<br>IPS ☐<br>NIPT ☐
Your other concerns ...	SIPS: ....<br>IPS: ....<br>NIPT:	★★★★★	SIPS ☐<br>IPS ☐<br>NIPT ☐

➢ 3) Considering my concerns and their importance, I choose ...

SIPS ☐ IPS ☐ NIPT ☐

© Université Laval, 2017. All rights reserved

➢ ***Are you comfortable with your decision?***			
**S**	1) Do you feel sure about the best choice for you?	Yes ☐	No ☐
**U**	2) Do you feel you have all the information you need to make a decision about prenatal screening test?	Yes ☐	No ☐
**R**	3) Are you clear about which benefits and harms matter most to you?	Yes ☐	No ☐
**E**	4) Do you have enough support and advice to make a choice?	Yes ☐	No ☐
**© SURE test : O'Connor et Légaré, 2008.**			

References:

Alldred et al. Cochrane lib. 2017. Badeau et al. Cochrane lib. (Under review). Hartwig et al. 2016; (36): 643-649. Schieve et. al. Disabil Health J. 2011; (4): 68–77. Alberta STE report, Aug. 2014. Graff et al. Am J Med Genet Part A.2015;167A:765-767. Akolekar et al. Ultrasound Obstet Gynecol. 2015 Jan; 45(1):16-26. Kleinveld JH et al. Prenat Diagn. 2006;26(4):354-61. ACOG Practice Bulletin No. 77. Obstet Gynecol. Jan 2007;109(1): 217-227. Morris et al. J Med Screen. 2002; 9(1): 2-6. Malone et al. N Engl J Med. 2005; 353(19): 2001-2011. Wald et al. Health Technol Assess. 2003; 7(11): 1-77. Green et al. Health Technol Assess. 2004; 8(33): iii, ix-x, 1-109. Won et al. Prenatal diagnosis. 2005; 25(7): 608-611

Authors:

Samira Abbasgholizadeh Rahimi (Eng, MHL, PhD) [1], Titilayo Tatiana Agbadje (MSc)[1], Jordie Croteau[1] (MSc)[1], Hubert Robitaille[1] (PhD), Mylène Badeau (Msc) [1], Denis d'Amours[1] (MD), Myriam Tremblay[1] (MD), François Rousseau[1] (MD, MSc), Sylvie Langlois[2] (MD), Anik Giguère[1] (PhD) and France Légaré[1] (MD, PhD). [1]Université Laval, Québec, QC, Canada. [2]University of British Colombia, Vancouver, BC, Canada.

No conflict of interest to declare:

The development of the tool was financed by a research grant from Genome Canada and Génome Québec. Neither the funding agency, the authors nor their affiliated institutions have any interest at stake in the decisions made by patients after using this decision aid.

Next Update:

May 2018.

© Université Laval, 2017. All rights reserved
