# Supplementary material for: An Analytical Mobile App for Shared Decision Making About Prenatal Screening: Protocol for a Mixed Methods Study
Source: JMIR Res Protoc. 2019 Oct 8;8(10):e13321. doi: 10.2196/13321 (PMC6913686; doi:10.2196/13321)
Supplement: Multimedia Appendix 2 [file resprot_v8i10e13321_app2.pdf]

Appendix 2

Questionnaire # 1

The purpose of the following questions is to assess the relative importance of the criteria for your decision about Down syndrome prenatal screening. Please carefully consider all descriptions for each criterion before rating their importance. First, select the criterion that is more important to you. Then, select its importance, as in the example below (Note: 1 is "Equal importance" and 9 "Extreme importance"; if A and B have equal importance to you, circle number 1).

Example

	A	B	How important is this factor to you? Rate from 1 to 9.								
1)	A (circled)	B	1	2	3	4 (circled)	5	6	7	8	9

Questionnaire (Example of questions)

In this table, circle which criteria (A or B) is more important to you when deciding about Down syndrome prenatal testing, and rate how important it is to you (1 is "Equal importance" and 9 "Extreme importance").

	A	B	How important is this factor to you? Rate from 1 to 9.								
1)	Waiting time for the results<br>A	Cost of the test<br>B	1	2	3	4	5	6	7	8	9
2)	Waiting time for the results<br>A	Detection rate of the test<br>B	1	2	3	4	5	6	7	8	9
3)	Waiting time for the results<br>A	Week of pregnancy that test will be taken<br>B	1	2	3	4	5	6	7	8	9
4)	Cost of the test<br>A	Detection rate of the test<br>B	1	2	3	4	5	6	7	8	9
